# Reduction of Seizure Occurrence from Exposure to Auditory Stimulation in Individuals with Neurological Handicaps: A Randomized Controlled Trial

**DOI:** 10.1371/journal.pone.0045303

**Published:** 2012-10-11

**Authors:** Mark Bodner, Robert P. Turner, John Schwacke, Christopher Bowers, Caroline Norment

**Affiliations:** 1 MIND Research Institute, Santa Ana, California, United States of America; 2 Department of Neurosurgery, Johns Hopkins University, Baltimore, Maryland, United States of America; 3 Department of Neurosciences, Pediatrics, Epidemiology & Biostatistics, Medical University of South Carolina, Charleston, South Carolina, United States of America; 4 Department of Epidemiology and Biostatistics, Medical University of South Carolina, Charleston, South Carolina, United States of America; 5 Department of Neurosciences, Medical University of South Carolina, Charleston, South Carolina, United States of America; University of Regensburg, Germany

## Abstract

**Background:**

The purpose of this work was to determine in a clinical trial the efficacy of reducing or preventing seizures in patients with neurological handicaps through sustained cortical activation evoked by passive exposure to a specific auditory stimulus (particular music). The specific type of stimulation had been determined in previous studies to evoke anti-epileptiform/anti-seizure brain activity.

**Methods:**

The study was conducted at the Thad E. Saleeby Center in Harstville, South Carolina, which is a permanent residence for individuals with heterogeneous neurological impairments, many with epilepsy. We investigated the ability to reduce or prevent seizures in subjects through cortical stimulation from sustained passive nightly exposure to a specific auditory stimulus (music) in a three-year randomized controlled study. In year 1, baseline seizure rates were established. In year 2, subjects were randomly assigned to treatment and control groups. Treatment group subjects were exposed during sleeping hours to specific music at regular intervals. Control subjects received no music exposure and were maintained on regular anti-seizure medication. In year 3, music treatment was terminated and seizure rates followed. We found a significant treatment effect (p = 0.024) during the treatment phase persisting through the follow-up phase (p = 0.002). Subjects exposed to treatment exhibited a significant 24% decrease in seizures during the treatment phase, and a 33% decrease persisting through the follow-up phase. Twenty-four percent of treatment subjects exhibited a complete absence of seizures during treatment.

**Conclusion/Significance:**

Exposure to specific auditory stimuli (i.e. music) can significantly reduce seizures in subjects with a range of epilepsy and seizure types, in some cases achieving a complete cessation of seizures. These results are consistent with previous work showing reductions in epileptiform activity from particular music exposure and offers potential for achieving a non-invasive, non-pharmacologic treatment of epilepsy.

**Trial Registration:**

Clinicaltrials.gov NCT01459692

## Introduction

Neurologically-impaired individuals may have significant neurologic morbidity related to epilepsy and seizure disorders. Finding safe, non-invasive methods of decreasing and preventing seizures is of paramount importance in improving the lives of those with epilepsy.

Epilepsy and seizures may arise through a variety of mechanisms [1]–[3]. It is well documented that brain stimulation from multiple sensory modalities [4]–[11] or internal cognitive processes [7], [8], [12]–[14] can induce seizures in predisposed individuals. Neurophysiological and clinical work, as well as theoretical and computational studies has indicated that certain stimuli or conditions may promote or evoke seizure which may be enhanced or “learned” [15]–[19], while in contrast, other specific stimuli may interfere with, or prevent seizures [18]–[31].

While most current types of neurostimulation are invasive, evidence has accumulated suggesting forms of noninvasive stimulation of the cortex by patterned external stimuli may be efficacious in reducing or preventing seizures. Specifically, stimulation of the cortex by exposure to particular patterned auditory stimuli (e.g. particular music) may reduce or even prevent or terminate epileptiform/seizure activity in many individuals [31]–[38]. Work by Hughes et al., [31] indicated an apparent anti-epileptogenic effect in a study exposing subjects to a Mozart Sonata (Sonata for 2 pianos in D Major, K. 448), with 23 of 29 subjects examined exhibiting significant decreases in epileptiform discharges. In another study by Turner [32], [33], it was reported that exposure to the K.448 stimulus resulted in significantly decreased interictal epileptiform discharges in subjects with Rolandic seizures. In a recent study by Lin et al., [36] it was indicated that specific components of that particular music stimulus reduced epileptiform discharges during and immediately after exposure to the K. 448 stimulus.

Subsequent studies employing the K.448 stimulus indicated a sustained effect might be accomplished through long-term exposure [34], [35], [37]–[39]. Hughes reported a potential long-term reduction in a case study through sustained exposure to the stimulus [39]. In another case study, Lahiri and Duncan [35] reported that long-term exposure to Mozart music (45 minutes per day over 3 months), resulted in a cessation of secondary generalized tonic-clonic seizures. Lin et al., [37], [38] reported long-term reduction in epileptiform discharge and seizures in both normal subjects and subjects with profound and severe levels of functioning who were exposed regularly to the Mozart Sonata K.448 over a 6 month period.

Studies of cortical activation from stimulation by music exposure have shown that music with different structures evokes differentially distributed and sustained excitation of cortical areas and rhythms [36], [40], [41]. Imaging and neurophysiological studies have shown that exposure to the K.448 stimulus evokes widely distributed persistent activation of cortical areas; particularly prefrontal cortex, but also including other frontal areas along with inferotemporal, occipital, and parietal areas of the cortex [40], [41]. Support for potential persistent effects from extended exposure has been attained in both animal models [42], [43] and humans [35], [37]–[39].

While active listening to the auditory stimulus may be more effective in initiating anti-epileptiform patterns of cortical activation, it has been indicated that activation can result also from passive exposure or even during certain sleep cycles [31], [32], [41], [44]. This suggests a protocol (used in the present study) in which subjects can be passively exposed (i.e. during sleep) for extended periods, making treatment less intrusive and more practical.

The apparent anti-seizure/anti-epileptiform effect of cortical stimulation from specific music (i.e. K.448) has not been subject to a randomized controlled clinical trial methodology. This formed the foundation of designing and performing the current clinical trial. In this study we examined the long-term effects of extended passive exposure to specific music (i.e. during sleep) on seizure frequencies of neurologically impaired individuals with epilepsy. The exposure is carried out during sleeping hours to enable long-term (1 year) extended exposure (over 10 hours nightly) of subjects to the treatment stimulus, which from the previous studies discussed above [31], [32], [39], [41], [44], can still efficaciously stimulate the cortex to facilitate a long-term effect. This study is the first to examine the effect specifically on seizure occurrence itself in a randomized controlled study. It also, to our knowledge examines the largest sample size of subjects examined for this phenomenon to date.

## Methods

The protocol for this trial and supporting CONSORT checklist are available as supporting information (see Checklist S1 and Protocol S1). The primary objective of this trial was to evaluate the efficacy of reducing the occurrence of seizures through cortical activation evoked from passive exposure (during sleep) to specific auditory stimulation (music—Mozart Sonata for 2-pianos, K.448) compared to control subjects receiving only regular antiepileptic drug (AED) treatment.

### Ethics

This block-randomized controlled study was conducted with IRB approval in the Medical University of South Carolina (MUSC). Since the majority of the individuals were unable to give informed consent, special language was included in the consent form for the parent/legal guardian acting on the individuals' behalf (SSDDSN, form 535-07-PD). Written informed consent was obtained from all participants involved in the study, or from the parent/legal guardian of participants whose capacity to consent was reduced.

### Participants

**Figure 1:**
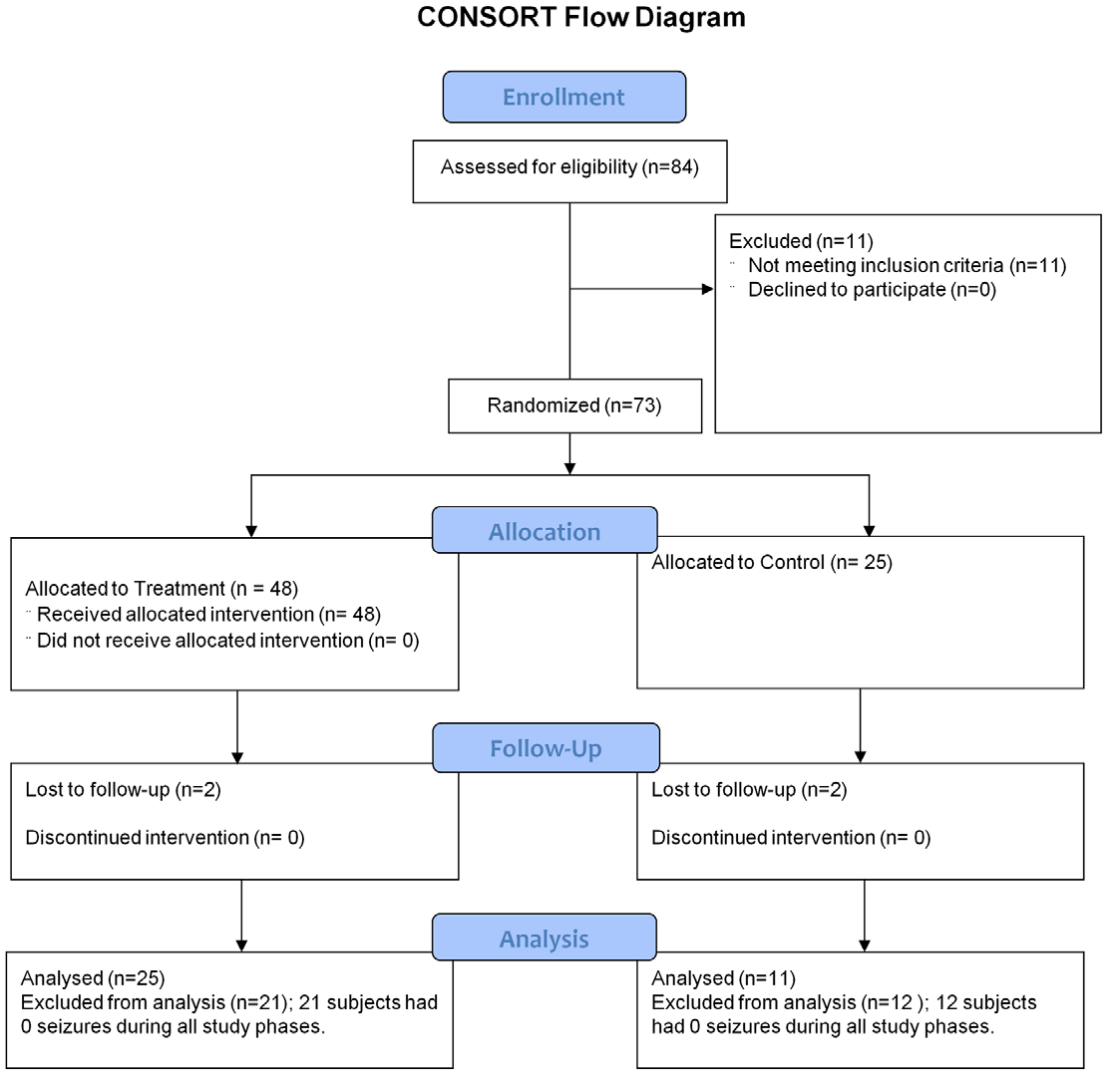
Patient Flow Diagram.

Subjects resided at the Thad E. Saleeby Center in Hartsville, SC, which is a permanent residence for individuals with heterogeneous neurological impairments, many with epilepsy or seizures related to an underlying disorder (Table 1). All individuals at the Saleeby Center were viewed as prospective candidates for study participation. The mean age of subjects was 36 years 11 months ±15 years 6 month (range 12 years to 78 years). Inclusion criteria consisted of: 1) resident of Thad E. Saleeby Center, 2) epilepsy or seizure disorder, 3) minimum one year detailed seizure reporting prior to study starting date (baseline seizure rate established), and 4) subjects exhibited seizures during any phase of the study to be included for final analysis. Exclusion criteria consisted of: 1) Subjects had no history of seizures, 2) Severe hearing impairment, 3) failure to obtain consent, and 4) subjects exhibited no seizures during any phase of the study. Seventy-three subjects were initially enrolled and 36 subjects completed the study and met the criteria for inclusion in final analysis (Figure 1).

**Table 1:** Distributions of treatment and control group subjects included in final analysis across variables of the study.

	Treatment	Control	Significance
**Gender**			
**Male**	11(48.1%)	5(46.2%)	*p* = 0.99
**Female**	14(51.9%)	6(53.8%)	
**Seizure Type**			
**Focal**	12(44.4%)	5(45.4%)	*p* = 0.99
**Generalized**	4(14.8%)	2(18.2%)	
**Focal and Gen.**	4(14.8%)	1(9.1%)	
**Gen. and Myoclonic**	5(18.5%)	3(27.3%)	
**Classification**			
**Idiopathic**	12(48.1%)	5(46.2%)	*p* = 0.72
**Symptomatic**	13(51.9%)	6(53.8%)	
**Neurological Impairment**			
**Cerebral Palsy**	8 (29.6%)	1 (15.4%)	*p* = 0.15
**Trisomy (14,18,21)**	2 (7.4%)	2 (15.4%)	
**Angelman Syndrome**	3 (11.1%)	1 (7.7%)	
**Anoxic Brain injury**	0 (0%)	1 (7.7%)	
**Rett Syndrome**	0 (0%)	1 (7.7%)	

The number of subjects in each category is given and the percentage of the group that it represents is indicated in parenthesis. Significance of the difference in the distributions of the groups is indicated on the right (2-sided Fisher Exact test). Note there are no significant differences between groups for any variable. The distribution of neurological impairments of symptomatic subjects is given at the bottom of the table.

Diagnosis of subjects was made and subjects were followed by the same epileptologist/pediatric neurologist during monthly clinics. Diagnostic EEGs were available with all subjects being followed.

### Randomization

Random assignment of subjects was carried out using a block-randomization based on a computer-generated algorithm. Seventy-three subjects satisfied the initial inclusion criteria for the study and were randomized to treatment and control groups. Block sizes consisted of 48 subjects for the treatment groups and 25 subjects for the control group. The study was intentionally unbalanced with approximately twice as many subjects in the treatment groups as the control. The 2∶1 ratio was performed to ensure that a sufficient number of treatment subjects remained in the study for analysis at the end of the post-treatment year, taking into account a potentially high rate of attrition of subjects from mortality or transfer (e.g. to home or another facility), as well as exclusion from final analysis due to exhibiting a complete absence of seizures throughout the study.

Randomization was carried out so as to minimize differences in baseline seizure frequency between the treatment and control groups, and was stratified according to level of functioning, age, and gender. Treatment and control groups were also randomized such that no significant differences were present between groups in seizure classification (idiopathic, symptomatic), or type (focal, generalized, focal and generalized, generalized and myoclonic)—Table 1.

Thirty-three subjects (21 treatment group, 12 control group) were excluded from final analysis for not meeting the inclusion criteria of exhibiting a least a single seizure during the study. Thirty-six subjects completed the study (Figure 1) and were included in final analysis (25 subjects in the treatment group, 11 subjects in the control group). Statistical analysis was carried out to take into account potential differences in study covariates (e.g. baseline seizure rates) between groups resulting from exclusion of non-seizure subjects from final analysis, and an unbalanced design (i.e. 2∶1 treatment group to control group subject ratio).

### Intervention

**Figure 2:**
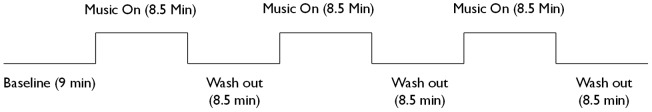
Protocol of music exposure administered to the treatment group every evening from 9:00 pm until 7:00 am. The sequence of exposure which was repeated consecutively 3 times each hour was: 1) 9 minute baseline period with no music, 2) K.448 played for 8.5 minutes (complete presentation of first movement), 3) 8.5 minute washout period with no music. The final washout period each hour (8.5 minutes) and the initial baseline period of the next consecutive hour (9 minutes) resulted in a 17.5 minute period of silence between the final music exposure of an hour and the first music exposure of the next hour.

The study treatment consisted of exposure to a specific auditory stimulus (music—Mozart Sonata for 2 pianos K.448) presented at periodic intervals (Figure 2) during the treatment year. Subjects in the treatment group were exposed to the stimulus protocol nightly during sleeping hours (9:00 pm to 7:00 am). The stimulus protocol of periodic presentation of K.448 was based on a similar sequence shown in previous imaging studies to evoke significant widely distributed and differential cortical activation patterns in subjects [41]. Presentation of the treatment stimulus was carried out during sleeping hours to enable long-term extended exposure (10 hours nightly over treatment year) which would not be practical during wakefulness. We wished to examine the impact of extended exposure since although the amount of exposure to achieve therapeutic results is not established, previous studies have indicated that extended exposure might achieve long-term persistent changes [34]–[40], [42], [43]. Further previous studies had indicated that passive exposure (i.e. during certain cycles of sleep) can still efficaciously stimulate the cortex to enable a long-term effect of extended exposure [32], [33], [39], [41], [44]. Nonetheless, presentation of the music began as subjects were beginning sleep and continued during the night through waking, so that some amount of exposure during wakefulness was received by the subjects.

The stimulus was delivered through a central sound system installed at the Saleeby center (with speakers in each subject's room) to ensure even exposure in treatment group subjects. A fixed music volume (approximately 60 db—normal conversational levels) was maintained through the exposure period. This volume ensured subjects received effective exposure but maintained a level that did not affect sleep—verified by an analysis of changes in waking events in participants measured between baseline and treatment years. The total number of seizure events for subjects were recorded during this phase. In the follow-up post-treatment year, stimulus exposure was stopped and seizure occurrences continued to be monitored. Control subjects received no exposure to the auditory stimulus during any phase of the study. All subjects in both treatment and control groups were maintained on their regular AED treatment during all phases of the study.

### Data Collection and Outcome Measures

The primary outcome measure of this study was to evaluate the effect of the music stimulus (K.448) on seizure frequency. Seizure events were determined through round-the-clock surveillance of subjects by the staff at the Saleeby Center, who are trained to observe seizure activity and to maintain detailed records of those events. Monthly clinics and continued staff education and training assisted in the identification of seizures and non-epileptic events. Data was also collected on hourly sleep patterns for analysis of potential changes occurring during different study phases which could affect seizure rates. The asleep or awake status for each subject in the study was monitored every hour during the night at the Saleeby Center, and the number of hours with instances of waking events was recorded. The outcome measure and sleep data were collected by the subject care staff according to the standard recording procedures in place at the Saleeby Center, and were extended to include all participants.

All attempts were made to avoid study bias by 1) subjects being blind to outcome measures, 2) only night-shift subject care staff were aware of the music treatment exposure (day-shift staff were blind to treatment and outcome measures), and 3) the same recording staff procedures of seizure activity and sleep activity were maintained throughout all phases of the study (Baseline, treatment, and post-treatment follow-up years).

Changes in seizure frequency across phases of the study were determined and statistically compared within the treatment group as well as between treatment and control groups to assess treatment efficacy. A post-hoc objective was to evaluate whether differential efficacy of treatment occurred as a function of study covariates (seizure classification and gender).

### Statistical Methods

Statistics were compiled on the number of seizure events for participants in the treatment and control groups during each phase of the study (Baseline, Treatment, Post-Treatment follow-up). The data was analyzed using regression methods appropriate for seizure count data and unbalanced designs. Specifically, a rate model was used to estimate the magnitude of the treatment effect and its association with patient covariates.

The rate model assumed that the observed seizure counts in the patients were Poisson distributed with rate parameter λ and that log(λ) is linear in the model effects. The model was fitted using maximum likelihood (generalized linear model) methods.

The baseline model is given by 
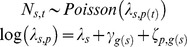
Where *N_s,t_* is the number of seizures observed for subject *s* in month *t*, *λ_s_* is the log baseline seizure rate (subject-specific intercept), *γ_g(s)_* is the log rate ratio between treatment and control groups, and *ζ_p,g(s)_* is the log ratio treatment effect. Model indices include subject *s*, month *t*, study phase *p* (baseline, treatment, post-treatment years), and treatment group g(s). The model was fitted using the GLM function with Poisson family and log link. Focus was on the phase-group interaction term *ζ_p,g(s)_* in the model described above.

Post-hoc analysis was conducted in an effort to determine the influence of gender and seizure class (idiopathic or symptomatic) on response to treatment. The model given above was expanded to include the effects for gender and class interactions with study phase within the treatment group. Models were built and analyzed using the methods described above.

An analysis of changes in subjects' sleep was also carried out to assess whether any significant differences had occurred within groups between study phases (2-sided Fisher exact test). The analysis was conducted on the number of waking events which were recorded at the Saleeby Center by the staff that monitored subjects each hour and maintain detailed records of those events.

## Results

Between February 1, 2005 and January 31, 2008, 73 subjects were enrolled in the study. Forty-eight and 25 patients were randomized to the treatment and control groups respectively. Two patients from the treatment group and 2 patients from the control group did not complete the study (Figure 1). Twenty-one subjects in the treatment group and 12 subjects in the control did not exhibit seizures during any phase of the study and were excluded from final analysis. Twenty-five treatment group subjects and 11 control group subjects were submitted to final analysis.

### Baseline Data

No significant difference in baseline seizure rates of treatment and control groups was present at study onset. Following exclusion of participant subjects not included in final analysis, the Treatment and Control groups exhibited a significant difference however in their baseline seizure rates (Table 2). Treatment group subjects exhibited between 2 and 93 seizures with a median of 13.5 seizures, while control group subjects exhibited between 2 and 134 seizures with a median of 14 seizures. No significant difference was present between treatment and control groups in seizure type, classification, or gender (Table 1).

**Table 2:** Summary of statistics of baseline seizure rates for treatment and control group subjects included in final analysis.

	N	Baseline Rate (Seizures/month)	Baseline Rate Ratio
**Treatment**	25	1.26	0.84 (*p* = 0.04)
**Control**	11	1.46	
**Male**	16	1.01	0.66 (*p* = 0.001)
**Female**	20	1.53	
**Symptomatic**	19	1.56	1.55 (*p* = 0.001)
**Idiopathic**	17	1.00	

Baseline rates were estimated using Poisson regression on the observed monthly seizure counts during the baseline year. While attempts were made to minimize differences, the treatment and control group subjects completing the study and included in final analysis differed in baseline seizure rates (rate ratio of 0.84, p = 0.04). This difference in baseline rate was accounted for in the statistical analysis in determining the presence of a treatment effect. Specifically, changes in seizures were determined relative to each group's respective baseline seizure rates, and thus no systematic error was introduced into determining treatment effects. Note that while significant differences in baseline seizures were present as a function of gender and seizure classification, no significant differences in treatment effect was present for these covariates.

### Treatment Phase Analysis

In the treatment group 20 patients (80%) exhibited decreases in seizures, 4 patients (16%) exhibited increases in seizures, and 1 subject (4%) exhibited no change in seizures. Six patients (24%) exhibited an absence of seizures in the treatment year. In the control group 4 subjects (36.4%) exhibited decreases in seizures, 5 subjects (45.5%) exhibited increases in seizures, and 2 subjects (18.1%) exhibited no change in seizures. Two patients (18.1%) exhibited an absence of seizures in the treatment year.

**Figure 3:**
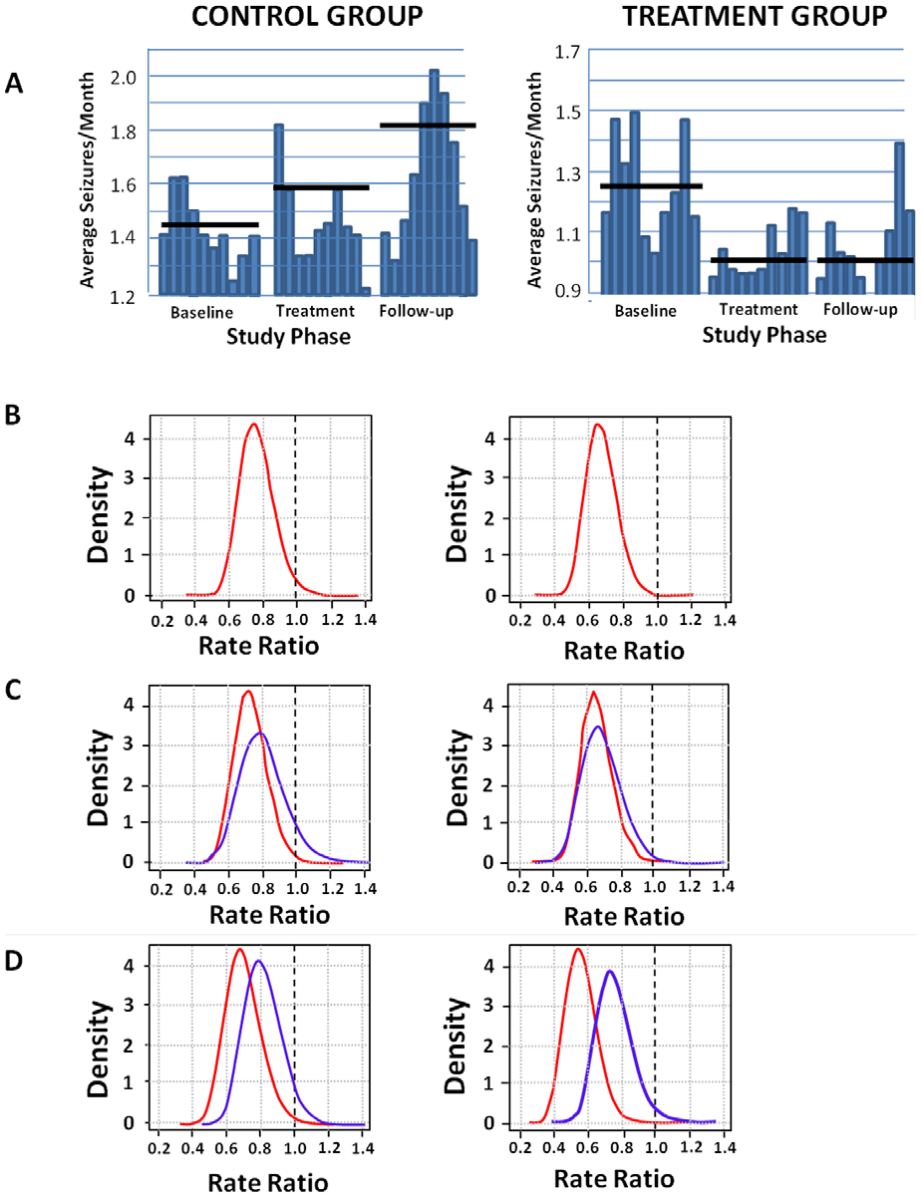
Seizure changes during study phases. A) Seizure rates across all phases of the study for the Control group (left) and Treatment group (right). Graphs show 3 month moving averages of seizure rates within each year, averaged across all subjects (i.e. first bar of the graph for each phase represents average seizure counts of months 1 through 3 of that phase, the second bar the average of months 2 through 4, and so on). The solid black horizontal lines indicate the average seizure rate within each phase. In the Control group the average seizure rate can be seen to increase in each consecutive year, while in the Treatment Group the seizure rate decreases from the baseline year rate, and maintains a reduced rate through the post-treatment follow-up year. B) Posterior densities for the treatment rate ratio in the treatment year (left) and in the follow-up year (right). The shift in the distribution of the treatment rate ratio (rate ratio = reduction in seizures in the treatment group/reduction in seizures control group) below 1.0 indicates the significant treatment effect in both the treatment and follow-up years. Posterior density was obtained using Markov Chain Monte Carlo methods and implemented using the rjags package [45] within the R computing environment [46]. The associated model reflects the model used in the reported GLM analysis. The smoothed plot was constructed by applying the R density function to 50,000 samples of the posterior. C) Posterior densities for the treatment rate ratio in the treatment year (left) for males (blue) and females (red) and in the post-treatment follow-up year (right). It can be seen from the graphs that no differential response from treatment was present as a function of gender. D) Posterior densities for the treatment rate ratio in the treatment year (left) for subjects with symptomatic seizures (blue) and idiopathic seizures (red) and in the post-treatment follow-up year (right). It can be seen from the graphs that while both groups of subjects exhibited a significant reduction in seizures, that subjects with idiopathic seizures exhibited a greater decrease in both treatment and follow-up years than those with symptomatic seizures.

The results of the rate model statistical analysis showed a significant (*p* = 0.024) decrease in seizures of 24% from baseline in the treatment group, in contrast to a 9.6% increase in seizures in the control group (Figure 3A and 3B). The estimated rate ratio due to treatment during the treatment phase is 0.76 with 95% confidence interval (0.599, 0.964)—Table 3A. These results indicate a clinically relevant response during the treatment year.

**Table 3:** Treatment effect from rate model analysis.

	GLM Estimate	Significance
**A. Treatment**	0.760 (0.599, 0.964)	*p* = 0.024
**Post Treatment**	0.671 (0.523, 0.859)	*p* = 0.002
**B. Treatment**	0.770 (0.600,0.980)	*P* = 0.033
**Post Treatment**	0.710 (0.550,0.910)	*P* = 0.006

A) Estimates of the coefficients from the GLM analysis focusing on the study phase interaction ζ_p,g(s)_ of the baseline rate model. Estimates are given with the 95% confidence intervals in parenthesis. The results indicate a significant treatment effect is present during the treatment phase (p = 0.024) and the post-treatment follow-up phase (p = 0.002). B) Estimates of the coefficients from the GLM analysis focusing on the study phase interaction ζ_p,g(s_ of the full model incorporating effects for gender and seizure class interactions with study phase within the treatment group. The results indicate a significant treatment effect is present during the treatment phase (p = 0.033) and the post-treatment follow-up phase (p = 0.006).

Analysis of the frequency of waking events indicated that no significant changes in sleep occurred from the baseline to the treatment year for either the treatment or control group (*p* = 0.39 2-sided Fisher Exact test).

### Post-Treatment Follow-up Analysis

The analysis from the rate model indicated a significant (*p* = 0.002) treatment effect with seizures decreasing 33% in the treatment group, in contrast to a 25.6% increase in seizures in the control group (Figure 3A and 3B). The estimated rate ratio due to treatment during the post-treatment phase is 0.671 with 95% confidence interval (0.523, 0.859)—Table 3A. The relatively large increase in the post-treatment phase average seizure rate for the control group could have inflated the treatment effect estimated by the model and thus warranted further consideration.

To take this into account, the study phase main effect was eliminated from the model (analyzing the change in seizures solely within the treatment group). This revealed that a statistically significant seizure rate reductions remained for both the treatment and post-treatment follow-up phases of 17% (p = 0.014) and 16% (p = 0.027) respectively. Thus the results indicate a clinically relevant response during treatment persisting one year post-treatment.

### Post Hoc Analysis

The rate model was expanded to include effects for gender and seizure classification (idiopathic, symptomatic) with study phase. An analysis of change in seizures in the expanded model (Table 3B) with study-phase and seizure class interaction added revealed a significant (p = 0.033) treatment effect during the treatment phase, persisting through the post-treatment follow-up phase (p = 0.006). The estimated rate ratio due to treatment during the treatment phase is 0.77 with 95% confidence interval (0.60, 0.98), and during the post treatment phase is 0.71 with 95% confidence interval (0.55, 0.91).

Within the treatment group, the effects of gender, either through its interaction with study phase alone, or in a three-way interaction with study phase and study group was not significant (Figure 3C). The effect of seizure classification through its interaction with study phase did appear significant in the model (p = 0.003). However, the three-way interaction of seizure class with study phase and group was marginal (p = 0.057). The associated model coefficient indicated a seizure rate increase of 55% for symptomatic subjects in the follow-up year which appears largely due to an increase among subjects in the control group. Reductions in seizures occurred in the treatment group for both symptomatic and idiopathic seizure classes in both treatment and post-treatment follow-up years (Figure 3D). However a greater decrease in average seizure rate was present for the idiopathic than for symptomatic subjects in both treatment and follow-up years.

## Discussion

Sustained passive exposure to specific music reduced seizure frequency in a significant percentage of neurologically impaired subjects with epilepsy. Seizure frequency decreased in the majority (80%) of subjects in the treatment group, whereas for control group subjects, over half exhibited either no change or increased seizure rates. Statistical analysis revealed a significant (p = 0.024) treatment effect existed, and that exposure to the auditory stimulation was likely to result in a seizure rate reduction of 24%. It should further be noted that 24% of treatment group subjects (6 subjects) exhibited a complete absence of seizures during treatment. Examining the long-term effects of treatment, it was observed that a significantly reduced average seizure rate of 33% was maintained after the termination of treatment during the post-treatment follow-up year. Thus the effect of extended treatment resulted in long-term reductions of seizure rates in the majority of subjects.

The results indicated that the reduction in seizures occurred across gender and seizure classification. However the decreases in subjects with idiopathic epilepsy appeared to be greater than those with symptomatic epilepsy. This is consistent with previous work in which decreases in epileptiform discharge significantly differed between subjects with idiopathic and symptomatic epilepsy in response to exposure to K.448 [38]. Also as in that study, no significant difference in seizure reduction was observed as a function of gender. With respect to seizure type, while the number of subjects was limited in the present study so that potential significant differences were not established, seizures were reduced in the majority of subjects across all seizure types (focal, generalized, focal and generalized, and generalized and myoclonic). Thus the observed therapeutic effect appeared to be a general effect.

The present study possessed limitations however, insofar as the number of participants was insufficiently large to enable an examination of potential differences in treatment effect as a function of seizure type or neurological handicap. Another possible confounding factor is that paroxysmal non-epileptic events may occur in the population. While such event occur infrequently, and randomization of the subjects in the study ensured that any such events, if present, would occur uniformly across groups so as to not bias the overall results, it is possible that such events could mask the efficacy of the treatment within given subgroups exhibiting specific seizure types—particularly for those types which occurred in small numbers of subjects. Further studies with larger populations are necessary to determine if the treatment might have any differential impact on specific different types of epilepsies or seizures.

It should also be noted that a small but significant difference in the average baseline seizure rate was present between treatment and control group subjects included in analysis. While it is possible that this difference could reflect some difference in the underlying source of seizures in the groups, this was accounted for by the statistical analysis which analyzed changes in seizures relative to each group respective baseline seizure rates, and thus no systematic error was introduced in the determination of the treatment effect.

The mechanism by which exposure to specific music reduces seizures is undetermined. However, studies have suggested possible mechanisms related to distributed and sustained cortical stimulation. Imaging studies of subjects during exposure to the K.448 stimulus [40], [41] revealed widely distributed cortical areas become activated (particularly prefrontal cortex, but also inferotemporal, occipital, and parietal cortex, along with other frontal areas), not consistently observed from exposure to other music or auditory stimuli. Though not necessarily unique in its effect, analysis of this music [36], [47], [48] has demonstrated that it has unique rhythmic structure and long-term coherence, which may account for its ability to stimulate widely distributed activation in the cortex and evoke particular rhythms with anti-epileptiform/anti-seizure properties. This auditory neurostimulation and its rhythmicity may account for the music's ability to decrease seizures, analogous to how other forms of neurostimulation (e.g. vagus nerve stimulation, deep brain/thalamic stimulation, etc.) may disrupt seizures. Computational studies have shown that neural networks biased towards seizure-like activity can be activated by stimuli with certain periodicities, while stimuli inducing other specific frequencies can disrupt or prevent seizure activity [15], [18], [30]. It is suggestive that exposure to the specific music has been reported to evoke short-term persistent activity in networks and cortical areas overlapping with those implicated in working memory [34], [40], [41] and to enhance or prime spatiotemporal reasoning ability in behavioral studies involving spatial- temporal working memory [42], [43], [49]–[52]. Particularly, strong activation of dorsolateral prefrontal cortex has been consistently reported to occur from the stimulation, which is an area critical in working memory, along with being an area possessing afferent and efferent connections with essentially the entire cortex [40], [41], [53]. Resonance in recurrent working memory networks, or disinhibition/hyperexcitability in such neuronal networks has been suggested to result in seizure activity [3], [18]. Thus the activation of such networks from exposure to the specific music could be indicative of “driving’ those networks in such a fashion that disrupts and/or inhibits seizure dynamics. From numerous studies it has been shown that music stimulation extends beyond auditory cortex involving a widely distributed neuronal network, which may explain the robust effect of the K.448 on epilepsy

This clinical trial protocol demonstrated the efficacy of treatment across a population with multiple neurological impairments and etiologies of seizures. However, while treatment was efficacious across all seizure classifications and types, the particular degree to which seizures were reduced was variable. Since all subjects had the same dose and duration of exposure, the variable effect of treatment may be attributed to inter-subject variability. Also, while the specific stimulus used in the present study might result in cortical activation in a range which is generally beneficial, other different stimulation parameters or frequencies may be more efficacious in particular subjects. Additionally, treatment exposure occurred primarily passively during sleep. The processing of the auditory stimulus and its resonance with cortical areas, although suggested in previous work to be similar in awake and at least specific cycles of sleeping states [31], [41], [44], may exhibit significant differences and be more efficacious with some degree of waking or active exposure. Indeed, recent work has suggested that active (waking) exposure may result in greater decreases in epileptiform activity than the corresponding decreases in seizures reported in the present study [37], [38]. Thus, while the passive exposure enables sustained treatment with potential long-term therapeutic effects, supplementing that with some amount of regularly administered, active wakeful exposure may prove even more efficacious. Future studies should examine larger numbers of subjects with similar types of seizures to answer these questions of optimization of dose/duration.

This work represents a step forward in the development of non-invasive treatments of epilepsy and seizures. Also, while the mechanism by which exposure to the K.448 stimulus decreased seizures in still under investigation, it should be studied as a potential treatment or add-on therapy for individuals with epilepsy and seizure disorder. Within the context of the computational and neurophsysiological studies, the existence of a positive treatment effect also represents a further step into understanding the epileptogenic process. This method of exposure to patterned auditory stimuli such as K.448, could lead to further understanding and research in epilepsy and its management [54].

## Supporting Information

Checklist S1 **CONSORT Checklist.**
(DOC)

Protocol S1 **Trial Protocol.**
(DOC)
