# Supplementary material for: Reduction of Seizure Occurrence from Exposure to Auditory Stimulation in Individuals with Neurological Handicaps: A Randomized Controlled Trial
Source: PLoS One. 2012 Oct 11;7(10):e45303. doi: 10.1371/journal.pone.0045303 (PMC3469625; doi:10.1371/journal.pone.0045303)
Supplement: Protocol S1 — Trial Protocol. (DOC) [file pone.0045303.s002.doc]

**Medical University of South Carolina**

**Protocol**

**PI Name:** **Robert P. Turner, MD, MSCR**

**Study Title**: **The Effect of Music Exposure on Seizure Frequency in Neurologically-Impaired Individuals**

*Once protocol is complete, save it as a Word document. Go back to the IRB application and upload the protocol.*

**TABLE OF CONTENTS –** *Prepare a table of contents based on the following outline, including page numbers, and insert here.*

** to be filled in after edits **

**A. SPECIFIC AIMS___________________________________________________________________________**

*List the broad, long-term objectives and the goal of the specific research proposed, e.g., to test a stated hypothesis, create a novel design, solve a specific problem, challenge an existing paradigm or clinical practice, address a critical barrier to progress in the field, or develop new technology.*

This study is a clinical trial investigating exposure of Mozart's Sonata for Two Pianos in D Major (hereafter K448) to a large cohort of pediatric and adult individuals with special needs, many of whom have epilepsy, who reside at the Thad E. Saleeby Center in Hartsville, SC. All individuals at the Thad E. Saleeby Center will participate in the study assuming completion of appropriate inclusion criteria, IRB approval from MUSC/MUHA and DDSN, and appropriate consent completion.

The specific aim of this study is to determine if exposure to K448 decreases seizure frequency in neurologically-impaired individuals. Detailed seizure-reporting is standard protocol at the Saleeby Center and therefore baseline data has been accumulated for the previous 4 years, making for long-term accumulation of pre- and post-exposure data analysis.

Secondary outcome measures will be collected, including (1) sleep patterns, (2) neurobehavioral dysfunction, and (3) other seizure characteristics. These outcome measures will be collected according to the standard recording procedures currently in place at the Saleeby Center, but will be extended to include all individuals.

**B. BACKGROUND AND SIGNIFICANCE_________________________________________________________**

*Briefly sketch the background leading to the present application, critically evaluate existing knowledge, and specifically identify the gaps that the project is intended to fill. State concisely the importance and health relevance of the research described in this protocol by relating the specific aims to the broad, long-term objectives. If the aims of the study are achieved, state how scientific knowledge or clinical practice will be advanced.*

Epilepsy is a world-wide disorder effecting all age and ethnic groups with substantial impact on quality of life, morbidity, and mortality. Current treatments focus on amelioration of symptoms, e.g. decreased seizures, but fail in curing epilepsy. Research is increasingly focusing on this goal of curing epilepsy. Finding safe, non-invasive methods of decreasing seizures, and potentially reversing the epileptogenic process, is of paramount importance in improving the lives of those with epilepsy.

**This study focuses on a specific and safe method of attempting to alter seizure frequency in persons with epilepsy. The generalized Mozart effect has documented short- and long-term effects in brain functioning, including decrease in seizures and epileptiform discharges (Turner 2004, Hughes 2001). The generalized Mozart effect has only been described within the last 15 years, and consists of enhanced or normalized higher brain function in response to exposure to music.**

**This effect was first published in the seminal study by Rauscher and colleagues (1993) who demonstrated enhanced spatial-temporal (ST) reasoning in a cohort of college students exposed to the Mozart Sonata for Two Pianos in D Major, K. 448 (hereafter K448). Patterned stimulation, exemplified in the Generalized Mozart effect, was initially studied in the 1980’s by Leng, McGrann, and Shaw who demonstrated reversal of the epileptic state based on the mathematical trion model of Mountcastle’s columnar organization of the mammalian neocortex. This ultimately led to demonstration of decreased seizures and interictal epileptiform discharges (IEDs) by Hughes in the 1990’s. Hughes work is currently being extended to systematic studies of the Generalized Mozart effect on the human EEG and IEDs (Turner 2004). The generalized Mozart effect has also been exhibited in animal models. A recent study at the M.I.N.D. Institute reveals enhanced spatial maze learning in rats exposed to K448 (Aoun in press).**

**Ideally, the proposed study will put a novel stimulus for studying epilepsy to the rigors of scientific testing using a large cohort of subjects. This method of exposure to K448, if effective, could lead to a new understanding of epilepsy and perhaps result in new forms of non-invasive seizure management.**

**C. PRELIMINARY STUDIES___________________________________________________________________**

*Provide an account of the principal investigator’s preliminary studies pertinent to this protocol and/or any other information that will help to establish the experience and competence of the investigator to pursue the proposed project.*

The principle investigator has conducted two preliminary studies for this project. The first, published in Epilepsy and Behavior, was a randomized, single-blinded, crossover, placebo-controlled, clinical trial investigating the effect of Mozart's Sonata for Two Pianos (K448) on the frequency of interictal epileptiform discharges (IEDs) in EEGs of children with benign childhood epilepsy. Two of the four subjects in the pilot study demonstrated sufficient waking IEDs for statistical analysis. Significant decreases in IEDs per minute (33.7, 50.6 and 33.9%) were demonstrated when comparing baseline with exposure to K448, but not to control music (Beethoven's Fur Elise).

**The second study was expanded to include ten children with rolandic epilepsy. Twelve hours of sleeping EEG were recorded for each child while Mozart and Beethoven alternated quietly in the background. The data is currently undergoing Independent Component Analysis (ICA) to statistically compare the three conditions: silence (baseline), Mozart and Beethoven (control). Preliminary results indicate that sleep patterns were not disturbed by the background music.**

**D. RESEARCH DESIGN AND METHODS (including data analysis) __________________________________**

*Describe the research design and the procedures to be used to accomplish the specific aims of the project. Include how the data will be collected, analyzed, and interpreted and specify what statistical methods will be used. Describe any new methodology and its advantage over existing methodologies. Discuss the potential difficulties and limitations of the proposed procedures and alternative approaches to achieve the aims. As part of this section, provide a tentative sequence or time-table for the project. Point out any procedures, situations, or materials that may be hazardous to personnel and the precautions to be exercised.*

Study Subjects: Individuals at the Thad E. Saleeby Center are all candidates for participation in this study. The ages will range from 5 to 90 years old.

Inclusion criteria:

• Resident of the Thad E. Saleeby Center

• Epilepsy or seizure disorder

• At least one year of detailed seizure reporting prior to study starting date

Exclusion criteria:

• Severe hearing impairment

Written informed consent will be obtained for all participants. If an individual is not capable of giving informed consent, a parent or legal guardian will be asked to act in the best interest of the patient in accordance with DDSN Departmental Directive 535-07-PD (see consent form for more details).

Children and adults who have given informed consent to participate in this study will be randomized into two groups: Group 1 (2/3 of participants) will be exposed to Mozart music at night and Group 2 (1/3 of participants) will be exposed to silence at night. Special care will be taken to make sure the audio levels for Group 1 do not disrupt their sleep cycles. Preliminary data from a pilot study at MUSC shows that background music does not alter sleep patterns.

Group 1 Protocol: Exposure to K448 will occur nightly between the hours of 9:00 p.m. and 7:00 a.m. Each room will have a sound system installed in it to deliver the stimuli. The stimuli will be presented in the same pattern as the 2002 ten-subject pilot study at MUSC:

Pre-exposure: The first 9 minutes of each hour will be silent.

Exposure: 8.5 minutes of K448 will follow the silent baseline.

Post-exposure: 8.5 minutes of silence will follow K448 acting as a washout.

The exposure and post-exposure epochs will play 3 times each hour before the cycle repeats the following hour beginning with another 9 minute baseline. Since the cycle is exactly 60 minutes in length, we will be able to deliver the entire stimulus train on one 80-minute compact disk from a central location in the Saleeby Center.

Group 2 Protocol: No music will be played for the control group. Evening and morning routines for both groups should remain identical.

Data Collection: A tentative start date for the project is January 1, 2006. Prior to this date, it is important to outline what data will be collected. The primary outcome measure for the study is seizure frequency. Therefore detailed records of seizure frequency by the Saleeby staff will be critical to quantifying any significant changes compared to baseline (previous years). Duration and intensity of seizures would also be useful as secondary measures of outcome in addition to sleep patterns and behavior.

Data will be collected on each individual for each outcome measure during the 12 month period of exposure. Interim analyses will occur monthly, quarterly, and at 6 months into the study.

**E. PROTECTION OF HUMAN SUBJECTS________________________________________________________**

1. RISKS TO THE SUBJECTS

*a. Human Subjects Involvement and Characteristics*

*- Describe the proposed involvement of human subjects.*

*- Describe the characteristics of the subject population, including their anticipated number, age range and health status.*

The proposed involvement of human subjects is limited to the exposure of Mozart music to a subset of subjects while they are asleep. Everything else pertaining to the daily routine of each subject will remain unchanged. The subject population all have disabilities and special needs and are permanent residents of the Thad E. Saleeby Center. The age range is large (approximately 5 to 90 years old). Many of the individuals have epilepsy or a seizure disorder and would possibly benefit from this study.

**Targeted/Planned Enrollment Table**

Total Planned Enrollment Will contact Sissy and Susan for these numbers…

| TARGETED/PLANNED ENROLLMENT: Number of Subjects | | | |
| --- | --- | --- | --- |
| **Ethnic Category** | Sex/Gender | | |
|  | Females | Males | Total |
| Hispanic or Latino |  |  |  |
| Not Hispanic or Latino |  |  |  |
| **Ethnic Category: Total of All Subjects*** |  | | |
| **Racial Categories** |  | | |
| American Indian/Alaska Native |  |  |  |
| Asian |  |  |  |
| Native Hawaiian or Other Pacific Islander |  |  |  |
| Black or African American |  |  |  |
| White |  |  |  |
| **Racial Categories: Total of All Subjects*** |  |  |  |

**The “Ethnic Category: Total of All Subjects” must be equal to the”Racial Categories: Total of All Subjects”.*

*- Identify the criteria for inclusion or exclusion of any subpopulation.*

*- Explain the rationale for the involvement of special classes of subjects, such as fetuses, neonates, pregnant women, children, prisoners, institutionalized individuals, or others who may be considered vulnerable populations. Note that 'prisoners' includes all subjects involuntarily incarcerated (for example, in detention centers) as well as subjects who become incarcerated after the study begins.*

*- If you propose to exclude any sex/gender or racial/ethnic group, include a compelling rationale for the proposed exclusion. For example, 1) the research question addressed is relevant to only one gender or 2) evidence from prior research strongly demonstrates no difference between genders.*

*- Provide either a description of the plans to include children or, if children will be excluded from the proposed research, then you must present an acceptable justification for the exclusion. For example, 1) the condition is rare in children as compared to adults or 2) insufficient data are available in adults to judge risk in children.*

*- List any collaborating sites where human subjects research will be performed, and describe the role of those sites in performing the proposed research.*

Thad E. Saleeby Center in Hartsville, South Carolina

b. Sources of Materials

*- Describe the research material obtained from living human subjects in the form of specimens, records, or data.*

*- Describe any data that will be recorded on the human subjects involved in the project.*

*- Describe the linkages to subjects, and indicate who will have access to subject identities.*

*- Provide information about how the specimens, records, or data are collected and whether material or data will be collected specifically for your proposed research project.*

The researchers working on this study will analyze the clinical information that is already routinely collected on the individuals at the Saleeby Center. This information includes observed seizures, sleep patterns and behavior. The researchers may also collect other information including the individuals medications as well as pretinent baseline information collected during the time prior to the study (e.g., seizure frequency, sleep patterns, behavior).

All of the data will be associated with a non-identifying number in place of the subject's name. Physical data will be locked up and electronic data will be password-protected. Only members of the research team will have access to this information.

c. Potential Risks

*- Describe the potential risks to subjects (physical, psychological, social, legal, or other), and assess their likelihood and seriousness to the subjects.*

*- Where appropriate, describe alternative treatments and procedures, including the risks and benefits of the alternative treatments and procedures to participants in the proposed research.*

There are minimal risks involved with this study. There is a slight chance that the music might disrupt a subject's sleep patterns. To prevent this from happening, the volume level of the Mozart music will be adjusted so that it does not wake them up or prevent them from falling asleep. Preliminary research at the Medical University of South Carolina by the principle investigator has shown that the music exposure does not affect a subject's sleep cycle.

2. ADEQUACY OF PROTECTION AGAINST RISKS

a. Recruitment and Informed Consent

*- Describe plans for the recruitment of subjects (where appropriate) and the process for obtaining informed consent. If the proposed studies will include children, describe the process for meeting requirements for parental permission and child assent.*

*- Include a description of the circumstances under which consent will be sought and obtained, who will seek it, the nature of the information to be provided to prospective subjects, and the method of documenting consent.*

Participants will be recruited from the current residents of the Thad E. Saleeby Center. Informed consent will obtained by the principle investigator, Dr. Robert Turner. Since the majority of the individuals are unable to give informed consent, special language has been included in the consent form for the parent/legal guardian acting on the individual’s behalf in accordance with the South Carolina Department of Disabilities and Special Needs (Form 535-07-PD).

b. Protection against Risk

- *Describe planned procedures for protecting against or minimizing potential risks, including risks to confidentiality, and assess their likely effectiveness.*

*- Where appropriate, discuss plans for ensuring necessary medical or professional intervention in the event of adverse effects to the subjects.*

*- Studies that involve clinical trials (biomedical and behavioral intervention studies) must include a description of the plan for data and safety monitoring of the research and adverse event reporting to ensure the safety of subjects in Section 4 below.*

The only change from normal care that is being proposed is that Mozart music will be quietly played in the background in one group of individuals. The data collection that will yeild the primary and secondary outcome measures is the same protocol that is already in place at the Saleeby Center. One possible risk is that an individual's sleep patterns may be distrubed. Preliminary studies at the Medical University of South Carolina by the principle investigator have shown that sleep cycles are not altered by the music. Special care will be taken to tailor the volume threshold on a patient-to-patient basis.

The risk of compromising protected health information (PHI) will be minimized by locking up physical medical records and password-protecting computer records. Only staff at the Thad E. Saleeby Center and members of the research team will have access to the research files.

3. POTENTIAL BENEFITS OF THE PROPOSED RESEARCH TO THE SUBJECTS AND OTHERS

*- Discuss the potential benefits of the research to the subjects and others.*

*- Discuss why the risks to subjects are reasonable in relation to the anticipated benefits to subjects and others.*

The potential benefit is that the music may decrease the frequency of an individual's seizures. The subjects' sleep patterns and behavior may also benefit from the exposure to Mozart. If this is the case, it is possible that this treatment could be used as a supplemental therapy for epilepsy. It is hoped that the information gained from this study will help researchers in the understanding and treatment of children and adults with epilepsy.

4. IMPORTANCE OF THE KNOWLEDGE TO BE GAINED

*- Discuss the importance of the knowledge gained or to be gained as a result of the proposed research.*

*- Discuss why the risks to subjects are reasonable in relation to the importance of the knowledge that reasonably may be expected to result.*

*- NOTE: Test articles (investigational new drugs, devices, or biologicals) including test articles that will be used for purposes or administered by routes that have not been approved for general use by the Food and Drug Administration (FDA) must be named. State whether the 30-day interval between submission of applicant certification to the FDA and its response has elapsed or has been waived and/or whether use of the test article has been withheld or restricted by the Food and Drug Administration, and/or the status of requests for an IND or IDE covering the proposed use of the test article in the research plan.*

The risk/benefit assessment for this project is excellent. The subjects are exposed to no more than minimal risk and the results, if valid, represent a significant non-invasive treatment for reducing seizure frequency, improving sleep patterns and reducing behavioral problems. Joining the research project is completely optional and if a subject joins and later decides to leave the study, they may do so without fear of penalty or loss of benefits.

5. SUBJECT SAFETY AND MINIMIZING RISKS (Data and Safety Monitoring Plan)

*Studies that involve *clinical trials (see description below) must include a description of the plan for subject safety and minimizing risks of the research, including data monitoring and adverse event reporting to ensure the safety of subjects. The complexity of the plan should be determined by the level of risk to subjects. The plan should specify: 1) what will be monitored, 2) how frequently the monitoring will occur, 3) who will be responsible for the monitoring, and 4) study endpoints.*

Data and safety monitoring will be the responsibility of the principle investigator (Dr. Robert Turner) and the Co-directors of the Thad E. Saleeby Center (Sissy Spann and Susan Moore).

1) The primary outcome that will be monitored will be seizure frequency. The staff at the Saleeby Center are trained to observe seizure activity and maintain a detail record of such events. The secondary outcome measures include sleep patterns and changes in behavior. At night, the staff is required to check on each patient every 30 minutes and record if they are asleep or not. Certain behavioral characteristics (screaming, hitting, nail-biting, crying, inappropriate undressing, etc.) are recorded for each individual.

2) The individuals are monitored around the clock. Data will be collected for one calendar year. Since the outcome measures that will be collected for the study are the same pieces of information that have already been recorded for their clinical management, a detailed baseline comparison can be made with previous years.

3) Data monitoring will be the responsibility of the staff at the Thad E. Saleeby Center. Seizures, sleep patterns and behavioral changes will be recorded as usual by the nurses. Specific behavioral measures will be monitored by the clinical psychologist, Eva Letki.

4) The study will end exactly one calendar year after it begins. The tentative start date is January 1, 2006.

*Clinical Trials

*A* clinical trial *is a prospective biomedical or behavioral research study of human subjects that is designed to answer specific questions about biomedical or behavioral interventions (drugs, treatments, devices, or new ways of using known drugs, treatments, or devices).*

*Clinical trials are used to determine whether new biomedical or behavioral interventions are safe, efficacious, and effective. Behavioral human subjects research involving an intervention to modify behavior (diet, physical activity, cognitive therapy, etc.) fits these criteria of a clinical trial. Human subjects research to develop or evaluate clinical laboratory tests (e.g. imaging or molecular diagnostic tests) might be considered to be a clinical trial if the test will be used for medical decision-making for the subject or the test itself imposes more than minimal risk for subjects.*

**F. REFERENCES/LITERATURE CITATIONS______________________________________________________**

*List all references. Each reference must include the title, names of all authors, book or journal, volume number, page numbers, and year of publication. The reference should be limited to relevant and current literature. It is important to be concise and to select only those literature references pertinent to the proposed research.*

Aoun P, Jones T, Shaw GL, Bodner M. Long-term enhancement of maze learning in mice via generalized

**Mozart effect. Neurological Research In Press.**

**Hughes JR. Review: The Mozart Effect. Epilepsy & Behavior 2001; 2(5):396-417.**

**Leng X, McGrann JV, Shaw GL. Reversal of epileptic state by patterned electrical stimulation suggested**

**by trion model calculations. Neurological Research 1992;14(1):57-61. UI: 1351261**

**Mountcastle VB. The columnar organization of the neocortex. Brain 1997;120:701-22. UI: 9153131**

**Rauscher FH, Shaw GL, Ky KN. Music and spatial task performance. Nature 1993; 365(6447):611. UI:**

**8413624**

**Turner RP. The acute effect of music on interictal epileptiform discharges. Epilepsy Behav. 2004 Oct;**

**5(5):662-8.**

G. CONSULTANTS__________________________________________________________________________

*Where applicable, attach electronic versions of appropriate letters from all individuals confirming their roles in the project. Go to the application under “additional uploads” to attach this information*.

H. FACILITES AVAILABLE____________________________________________________________________

*Describe the facilities available for this project including laboratories, clinical resources, etc.*

The Thad E. Saleeby Center is a state-run facility operating under the South Carolina Department of Disabilities and Special Needs. The Saleeby Center is a permanent residence home for individuals with specials needs and disabilities who require assisted living. There are fifty-five 2-patient bedrooms divided among three wings of the facility. There are also 14 unique day rooms that are specifically designed for different tasks including reading, arts and crafts, physical therapy, etc. The Co-directors of the Saleeby Center stress a high level of care tailored specifically to each individual’s unique needs. There are currently 85-92 individuals residing at the Saleeby Center, most of whom have epilepsy or a seizure-related disorder and might benefit from this research study.

I. INVESTIGATOR BROCHURE ____________________________________________________

*If applicable, attach the electronic version of the investigator brochure. Go to the application under “additional uploads” to attach this information.*

J. APPENDIX_______________________________________________________________________________

*Attach any additional information pertinent to the application, such as surveys or questionnaires, diaries or logs, etc. Go to the application under “additional uploads” to attach this information.*
